# Drivers of phenotypic divergence in a Mesoamerican highland bird

**DOI:** 10.7717/peerj.12901

**Published:** 2022-02-18

**Authors:** Sahid M. Robles-Bello, Melisa Vázquez-López, Sandra M. Ramírez-Barrera, Alondra K. Terrones-Ramírez, Blanca E. Hernández-Baños

**Affiliations:** 1 Facultad de Ciencias, Biología Evolutiva, Universidad Nacional Autónoma de México, Ciudad de México, CDMX, México; 2 Posgrado en Ciencias Biológicas, Universidad Nacional Autónoma de México, Ciudad de México, CDMX, Mexico

**Keywords:** Morphometric variation, Genetic variation, *Piranga bidentata*, Cardinalidae, Carotenoid, Plumage color

## Abstract

Animals derive their coloration from a variety of pigments as well as non-pigmentary structural features. One of the most widespread types of pigments are carotenoids, which are used by all invertebrate taxa and most vertebrate orders to generate red, pink, orange and yellow coloration. Despite their widespread use by diverse animal groups, animals obligately obtain carotenoid pigments from diet. Carotenoid-based coloration is therefore modulated by evolutionary and ecological processes that affect the acquisition and deposition of these pigments into tegumentary structures. The Flame-colored Tanager (*Piranga bidentata*) is a highland songbird in the cardinal family (Cardinalidae) that is distributed from Mexican sierras through Central America up to western Panama. While female plumage throughout its entire range is predominantly yellow, males exhibit a noticeable split in ventral plumage color, which is bright orange on the West slope and the Tres Marias Islands and blood red in Eastern Mexico and Central America. We used Multiple Regression on Matrices (MRM) to evaluate the relative contributions of geographic distance, climate and genetic distance on color divergence and body differences between geographically disjunct populations. We found that differentiation in carotenoid plumage coloration was mainly explained by rainfall differences between disjunct populations, whereas body size differences was best explained by variation in the annual mean temperature and temperature of coldest quarter. These results indicate that climate is a strong driver of phenotypic divergence in *Piranga bidentata*.

## Introduction

The variety of colors in animal integuments is perhaps one of the most striking features of the natural world. These colorations serve a variety of signaling functions, as well as non-signaling functions such as camouflage (Land & Nilsson, 2012; Bortolotti, 2006). The enormous range of animal colors we observe is derived from the complex interplay of selective light absorption by chemical pigments, structural features that affect light scattering (for example the nanostructure of butterfly wing scales and bird feathers) and other processes such as blood irrigation of exposed fleshy areas (Shawkey & D’Alba, 2017; Shawkey, Morehouse & Vukusic, 2009). Carotenoids are used by almost every vertebrate order and all invertebrate phyla to generate yellow, orange, pink and red coloration (McGraw, 2006). However, how the evolution of carotenoid-based pigmentary coloration has resulted in the colors we see at present is unclear in many taxa. Carotenoids are unique among animal pigments in that they are obtained exclusively from diet, as animals lack the metabolic routes to synthesize them *de novo* (Britton, 1983). In many cases, dietary carotenoids undergo chemical modifications such as ketolation before being deposited in tissues (Lopes et al., 2016; McGraw, 2006; Hill, 2000). In many vertebrates such as birds and reptiles, carotenoid-based coloration is often considered to be used as an honest signal of individual quality (Zahavi, 1975; Olson & Owens, 1998; Steffen, Hill & Guyer, 2010; Jacot et al., 2010). Since they are obtained from diet and metabolically modified before deposition, carotenoids are thought to signal a high-quality diet, as well as high immune and metabolic function in a way that cannot easily be faked (Steffen, Hill & Guyer, 2010; Johnson & Hill, 2013).

Individuals that display more elaborate signals and brighter colors are thought to incur costs associated with these signals (Faivre et al., 2003; Martínez-Padilla et al., 2010). Since carotenoid molecules readily accept electrons, they function as antioxidants and carotenoid intake is related to body maintenance (Butler & McGraw, 2010) and immune function (McGraw & Ardia, 2003); carotenoid molecules that are locked away in metabolically inactive tissue such as feathers or scales are no longer available, and their modification and deposition diverts energetic resources away from body maintenance and direct reproductive effort (Hill, 2000). In addition, increasing the elaborateness of displays increases risk of predation, and in some cases, can increase parasite loads (Baker & Parker, 1979). It is likely that decreases in ornamentation such as the limitation of carotenoid-based signals such as having red coloration on only one part of the body or having reduced color intensity have evolved as a result of trade-offs between sexually selected signaling and survival-related natural selection (Hamilton & Zuk, 1982; Aguilera & Amat, 2007; Alonso-Alvarez et al., 2008).

Unlike melanin-based coloration, where the genetic and metabolic basis of different phenotypes is relatively well understood, we know remarkably little of the mechanistic basis of carotenoid coloration outside of model organisms (Jacot et al., 2010). This complicates the task of understanding which processes underlie divergence in coloration in species that derive their coloration from carotenoids. This difference might be caused by variation of the source of carotenoids available in the environment (Grether, 2000), genetically determined differences in the metabolic conversion and pigment deposition in integument tissues (Mundy et al., 2016), or direct selection based on environmental factors such as light availability and background color (Zink & Remsen, 1986) or abundance of feather-degrading microorganisms (Burtt Jr & Ichida, 2004). The mechanisms underlying these hypotheses are not mutually exclusive, and they are expected to overlap to some extent in most systems.

This variety of mechanisms modulating color expression nevertheless result in some well-known spatial patterns such as Gloger’s rule (Gloger, 1833), which predicts that endotherms living in more humid climates tend to be darker in coloration.

A study system in which closely related populations differ in the extent or intensity of their carotenoid-based pigmentary coloration would allow us to disentangle the relative contributions of these multiple selective pressures, as well as other non-selection processes such as genetic drift. Since carotenoid metabolism and deposition appear to be regulated by a large network of genes (Price-Waldman & Stoddard, 2021), if differences in color are due to fixed genetic differences between populations, we would expect to find some correlation between differences in the expressed color and genetic distance.

Similarly, changes in body size among populations can result from interactions among ecological factors such as food availability, genetic factors and other constraints such as thermoregulation efficiency. While it is intuitive that body size can evolve as a result of direct selection pressures, examples have been observed where the evidence for intraspecific changes in body size being caused by local adaptation is weak (Seeholzer & Brumfield, 2018). Both color and body size are complex traits that are affected by a number of interacting factors, and teasing apart the relative effects of climate, genetics and geographic distance is a complex problem. Simultaneous analysis of multiple sets of explanatory variables is necessary to better understand the processes that drive phenotypic divergence. With current statistical methods, we can explicitly test whether observed divergence in a trait is related to geographic distance (Isolation by distance, IBD), genetic changes, climate, or a combination of these factors.

**Figure 1:**
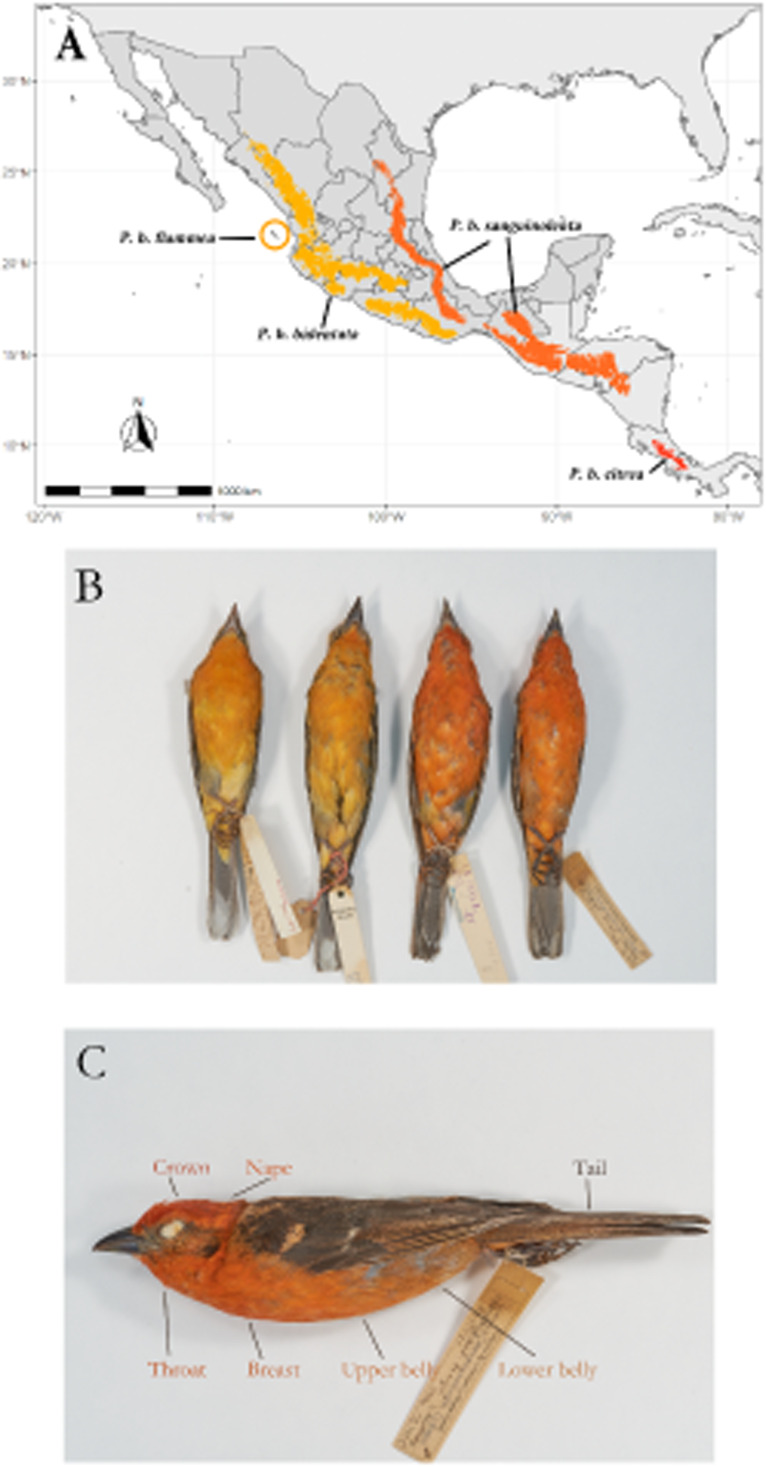
Geographic range of the species with subspecies labeled. Overview of the study system. (A) Geographic range of the species, with the subspecies ranges labeled. (B) Color differences between described subspecies. From left to right, *Piranga bidentata flammea*, *P.b. bidentata*, *P.b. sanguinolenta* and *P.b. citrea*. Subspecies descriptions following Howell & Webb; *P. bidentata bidentata*: Bill gray. Head and underparts flaming orange with dusky auriculars. Upperparts dusky orange, often washed olive, heavily streaked black on back. Wings and tail blacked, edged orangish with two broad whitish wingbars and bold white spots on tip of tertials and outer rectrices.*P. b. flammea*: Like bidentata, but underparts paler orange. Described on basis of disjunct geographic range. *P. b sanguinolenta*: Like bidentata, but has head and underparts red to orange red, wingbars and tertial spots often tinged red. *P. b. citrea*. Like sanguinolenta, underparts bright red. Described on basis of disjunct geographic range. (C) Feather patches from which we measured color.

The Flame-colored Tanager (*Piranga bidentata*) inhabits deciduous forests in the highlands of both of the major mountain systems in Mexico, as well as Central America and the mountains of west Panama from around 800 m.a.s.l. to the tree line (Fig. 1A). There is also an isolated population on the Islas Tres Marias off the Pacific coast of Mexico, which occupies dry shrubland and oak forest. Currently there are four recognized subspecies, described on the basis of a disjunct geographic distribution and differences in male plumage color (Howell & Webb, 1995; Hilty, 2020). This species shows marked geographic variation, with males on the Pacific side of the continent showing orange plumage while males on the Atlantic side (including Central America) show bright red coloration (Fig. 1B).

In this study, we used regression models to explore the phenotypic variation in plumage coloration and body size in *Piranga bidentata* and to look for relationships between this variation and environmental factors. Since this species is non-migratory exhibits largely isolated populations on a large geographic scale, we can use it as a model system to test for isolation by distance or local adaptation. We used both simple linear regression modeling and multiple regression on matrices (MRM; Lichstein, 2007) implemented in the R package *ecodist* (Goslee & Urban, 2007) with color distance as a response variable to test the relative contributions of geographic distance, genetic distance, distance in climate variables and differences in tree cover on the phenotypic variation in color as to distinguish between explanatory processes. We tested three different hypotheses about the factors that contribute to color variation in *Piranga bidentata*. First, if variation in color reflects phylogenetic relationships between subspecies, we would expect a strong relationship between divergence in color and genetic distance. Second, if plumage color is the result of local adaptation, ecological processes or phenotypic plasticity we would expect a strong relationship between divergence in color and climatic variable divergence. Finally, if color divergence is the result of the fixation of neutral alleles due to drift and reduced gene flow due to geographic distance, the isolation by distance model should be supported (IBD). Under this last model, we would expect a relationship between plumage color divergence and geographic distance. For body size, if climate is the proximate driver of divergence through selection on thermal constraints, we would expect a negative relationship between body size and mean annual temperature.

## Materials and Methods

### Genomic data

We extracted genomic DNA from 18 frozen tissue samples collected between 1991 and 2016 (*P. b. bidentata*: 2♂, *P. b. citrea*: 2 unknown sex, *P. b. flammea*: 1♀6♂, *P. b. sanguinolenta*: 4♀3♂; Table 1, Shown in a map in Fig. S1) from the MZFC tissue collection, as well as samples donated by other institutions, using the Qiagen DNeasy (Valencia, USA) kit protocol, the EPICENTRE MasterPure kit protocol as well as standard phenol-chloroform extraction. Samples deposited at the MZFC collection were obtained under a field collection permit provided by Instituto Nacional de Ecología, SEMARNAT, Mexico (FAUT-0169). We included two *Piranga roseogularis* and one *Cardinalis cardinalis* sample to act as outgroups in phylogenetic analyses. nextRad genotyping-by-sequencing libraries were obtained from our genomic DNA samples following the procedure described in Russello et al. (2015) at the SNPsaurus laboratory in the University of Oregon. Genomic DNA was first fragmented with Nextera reagent (Illumina, Inc.), which also ligates short adapter sequences to the ends of the resulting fragments. Since some of the samples had a high amount of degraded DNA, the Nextera reaction was scaled for fragmenting 20 ng of genomic DNA but 40 ng of genomic DNA was used for input to compensate, as well as to increase fragment sizes. Fragmented DNA was selectively amplified for 27 cycles at 74 degrees, with one of the primers matching the adapter and extending 10 nucleotides into the genomic DNA with the selective sequence GTGTAGAGCC. The nextRAD libraries were sequenced on a HiSeq 4000 platform with one lane of single-end 150 bp reads at an average depth of 54× (University of Oregon). Raw sequence reads are available at GenBank SRA (BioProject accession PRJNA757783).

**Table 1:** Voucher data. Specimens information data: Id, voucher, sex, subspecies, dates of collected, locality, latitude, longitude and data collected.

Museum	Voucher number	Sex	Subspecies	Collection date (DD/MM/YYYY)	Locality	Latitude	Longitude	Morphology data	Color data	Genomic data
MZFC	AGNS 0648	M	*Sanguinolenta*	15/09/1897	Mexico: Oaxaca, Metates, km 65 carretera Tuxtepec-Oaxaca	17.3633333	−96.5083333	X		
MZFC	AGNS 0649	M	*Sanguinolenta*	15/09/1897	Mexico: Oaxaca, Metates, km 65 carretera Tuxtepec-Oaxaca	17.3633333	−96.5083333	X		
MZFC	BMM 132	M	*Bidentata*	17/11/1989	Mexico: Michoacan, Pico de Tancítaro, 3 km N Zirimóndiro	19.3966667	−102.338333	X		
MZFC	BMM 220	M	*Sanguinolenta*	10/01/1990	Mexico: Oaxaca, Sierra Miahuatlán, Río Salado 10 km N San Gabriel Mixtepec	16.1	−97.1833333	X		
MZFC	BMM 654	F	*Sanguinolenta*	13/06/1991	Mexico: Hidalgo, 5 km E Tlanchinol	20.985	−98.6066667	X	X	X
MZFC	BMM 678	M	*Sanguinolenta*	14/06/1991	Mexico: Hidalgo, 5 km E Tlanchinol	20.985	−98.6066667	X		
MZFC	BMM 722	F	*Sanguinolenta*	15/07/1991	Mexico: Queretaro, 7 km S Tres Lagunas	21.2766667	−99.125	X		
MZFC	CAON 059	ND	*Bidentata*	19/08/1993	Mexico: Guerrero, Yetepetitlán	17.5539847	−98.9951969	X		
MZFC	CONACYT 0443	M	*Sanguinolenta*	13/12/2000	Mexico: San Luis Potosi, San Nicolás de los Montes, La Mesa	22.1216667	−99.425	X		
MZFC	DEUT 40	M	*Bidentata*	08/12/2000	Mexico: Michoacan, La Verdura	19.6683333	−102.545	X	X	X
MZFC	DEUT 41	M	*Bidentata*	08/12/2000	Mexico: Michoacan, La Verdura	19.6683333	−102.545	X		
MZFC	FD 193	M	*Bidentata*	03/05/1991	Mexico: Estado de México, km 14 de la carretera Ocuilan-Cuernavaca	18.9716667	−99.2933333	X		
MZFC	FD 198	M	*Bidentata*	02/05/1991	Mexico: Estado de México, km 14 de la carretera Ocuilan-Cuernavaca	18.9716667	−99.2933333	X		
MZFC	FD 201	F	*Bidentata*	20/05/1991	Mexico: Estado de México, km 15 de la carretera Ocuilan-Cuernavaca	18.9633333	−99.285	X		
MZFC	FD 227	M	*Bidentata*	14/06/1991	Mexico: Estado de México, km 14 de la carretera Ocuilan-Cuernavaca	18.9716667	−99.2933333	X		
MZFC	FRG 79	F	*Bidentata*	05/06/1981	Mexico: Nayarit, La Yerba, 11 km SW Tepic	21.4383333	−104.995	X		
MZFC	HGO-SLP 104	M	*Sanguinolenta*	02/03/1999	Mexico: Hidalgo, Cerro Jarros, 1 Km E El Sotano	20.9983333	−99.145	X	X	
MZFC	ITM 141	F	*Flammea*	21/03/2006	Mexico: Nayarit, Islas Marías, Isla María Madre, campamento El Zacatal	21.7402778	−106.654722	X		
MZFC	ITM 146	M	*Flammea*	21/03/2006	Mexico: Nayarit, Islas Marías, Isla María Madre, campamento El Zacatal	21.7402778	−106.654722	X		X
MZFC	ITM 156	M	*Flammea*	22/03/2006	Mexico: Nayarit, Islas Marías, Isla María Madre, campamento El Zacatal	21.7402778	−106.654722	X		
MZFC	ITM 159	M	*Flammea*	22/03/2006	Mexico: Nayarit, Islas Marías, Isla María Madre, campamento El Zacatal	21.7402778	−106.654722	X	X	X
MZFC	ITM 160	M	*Flammea*	21/03/2006	Mexico: Nayarit, Islas Marías, Isla María Madre, campamento El Zacatal	21.7402778	−106.654722	X	X	X
MZFC	ITM 188	M	*Flammea*	24/03/2006	Mexico: Nayarit, Islas Marías, Isla María Madre, campamento El Zacatal	21.7402778	−106.654722	X	X	X
MZFC	ITM 200	M	*Flammea*	23/03/2006	Mexico: Nayarit, Islas Marías, Isla María Magdalena, 2 do Campamento (aguada)	21.468781	−106.440783	X	X	X
MZFC	ITM 208	M	*Flammea*	28/03/2006	Mexico: Nayarit, Islas Marías, Isla María Madre, campamento El Zacatal	21.7402778	−106.654722	X		
MZFC	ITM 211	F	*Flammea*	28/03/2006	Mexico: Nayarit, Islas Marías, Isla María Madre, campamento El Zacatal	21.7402778	−106.654722	X	X	X
MZFC	ITM 223	M	*Flammea*	28/03/2006	Mexico: Nayarit, Islas Marías, Isla María Madre, campamento El Zacatal	21.7402778	−106.654722	X		
MZFC	ITM 233	M	*Flammea*	28/03/2006	Mexico: Nayarit, Islas Marías, Isla María Madre, campamento El Zacatal	21.7402778	−106.654722	X		
MZFC	ITM 240	F	*Flammea*	28/03/2006	Mexico: Nayarit, Islas Marías, Isla María Madre, campamento El Zacatal	21.7402778	−106.654722	X		
MZFC	ITM 245	M	*Flammea*	28/03/2006	Mexico: Nayarit, Islas Marías, Isla María Madre, campamento El Zacatal	21.7402778	−106.654722	X		
MZFC	ITM 246	M	*Flammea*	28/03/2006	Mexico: Nayarit, Islas Marías, Isla María Madre, campamento El Zacatal	21.7402778	−106.654722	X	X	X
MZFC	JEMP 062	M	*Bidentata*	12/06/1986	Mexico: Guerrero, Km 26.5 Carretera Taxco-Ixcateopan	18.51	−99.7566667	X		
MZFC	JEMP 0284	F	*Bidentata*	15/12/1986	Mexico: Guerrero, El Huizteco, 4 km NE Taxco	18.5666667	−99.6	X		
MZFC	JEMP 0285	M	*Bidentata*	15/12/1986	Mexico: Guerrero, El Huizteco, 4 km NE Taxco	18.5666667	−99.6	X		
MZFC	JEMP 0364	M	*Bidentata*	24/04/1987	Mexico: Guerrero, El Huizteco, 4 km NE Taxco	18.5666667	−99.6	X		
MZFC	JEMP 0419	F	*Bidentata*	07/05/1987	Mexico: Guerrero, El Huizteco, 4 km NE Taxco	18.5666667	−99.6	X		
MZFC	JK04 069	M	*Bidentata*	12/01/2004	Mexico: Guerrero, Carrizal de Bravo	17.816713	−99.967595	X		
MZFC	KABS 736	M	*Bidentata*	08/04/1993	Mexico: Nayarit, El Cuarenteño S. S. J.	21.4916667	−105.086667	X		
MZFC	PEP 208	ND	*Bidentata*	19/04/1982	Mexico: Nayarit, Venustiano Carranza, 10 km SW Tepic	21.5166667	−104.991667	X		
MZFC	PEP 218	F	*Bidentata*	19/04/1982	Mexico: Nayarit, Venustiano Carranza, 10 km SW Tepic	21.5166667	−104.991667	X		
MZFC	PEP 1268	F	*Sanguinolenta*	24/06/1987	Mexico: Hidalgo, Laguna Atezca, 4 km N Molango	20.805	−98.7466667	X		
MZFC	QRO 0298	M	*Sanguinolenta*	18/04/1997	Mexico: Queretaro, El Pemoche	21.2263056	−99.1096944	X	X	X
MZFC	QRO 0309	M	*Sanguinolenta*	19/04/1997	Mexico: Queretaro, El Pemoche	21.2263056	−99.1096944	X		
MZFC	RAY11 078	M	*Sanguinolenta*	05/10/2011	Mexico: San Luis Potosi, Gamotes, La Chicharrilla	21.79095	−99.524	X		
MZFC	RAY11 199	M	*Sanguinolenta*	10/06/2012	Mexico: San Luis Potosi, La Chicharrilla a 2 km al Oeste	21.8045833	−99.53695	X		
MZFC	SIN 067	M	*Bidentata*	31/01/1999	Mexico: Sinaloa, Rancho Mojocoan, 4km oeste de Copala	23.4027778	−105.901667	X	X	X
MZFC	TEPE 59	M	*Sanguinolenta*	14/05/2003	Mexico: Hidalgo, Texcapa	21.0933333	−98.8533333	X	X	X
MZFC	TEPE 60	F	*Sanguinolenta*	14/05/2003	Mexico: Hidalgo, Texcapa	21.0933333	−98.8533333	X	X	X
AMNH	40829	F	*Sanguinolenta*	ND	Guatemala?	ND	ND	X	X	
AMNH	68601	M	*Sanguinolenta*	01/04/1897	Mexico: Verzacruz, Jalapa	19.53124	−96.91589	X	X	
AMNH	68603	M	*Sanguinolenta*	01/04/1897	Mexico: Verzacruz, Jalapa	19.53124	−96.91589	X	X	
AMNH	77866	F	*Citrea*	26/08/1901	Panama: Chiriqui Province, Boquete	8.78024	−82.44136	X	X	
AMNH	77867	F	*Citrea*	05/09/1901	Panama: Chiriqui Province, Boquete	8.78024	−82.44136	X	X	
AMNH	77868	F	*Citrea*	05/09/1901	Panama: Chiriqui Province, Boquete	8.78024	−82.44136	X		
AMNH	77870	F	*Citrea*	07/09/1901	Panama: Chiriqui Province, Boquete	8.78024	−82.44136	X	X	
AMNH	77871	F	*Citrea*	08/09/1901	Panama: Chiriqui Province, Boquete	8.78024	−82.44136		X	
AMNH	77872	F	*Citrea*	14/09/1901	Panama: Chiriqui Province, Boquete	8.78024	−82.44136	X	X	
AMNH	77878	M	*Citrea*	03/09/1901	Panama: Chiriqui Province, Boquete	8.78024	−82.44136	X	X	
AMNH	77879	M	*Citrea*	06/09/1901	Panama: Chiriqui Province, Boquete	8.78024	−82.44136	X	X	
AMNH	77880	F	*Citrea*	07/09/1902	Panama: Chiriqui Province, Boquete	8.78024	−82.44136	X		
AMNH	84790	M	*Sanguinolenta*	06/06/1889	Mexico: Nuevo Leon, Camp 3 Boquillo	25.364444	−100.405278	X	X	
AMNH	84795	F	*Sanguinolenta*	07/06/1889	Mexico: Nuevo Leon, Camp 3 Boquillo	25.364444	−100.405278	X	X	
AMNH	84796	F	*Sanguinolenta*	10/05/1889	Mexico: Nuevo Leon, Camp 2 San Pedro Mines	25.364444	−100.405278	X	X	
AMNH	84797	M	*Sanguinolenta*	10/05/1889	Mexico: Nuevo Leon, Camp 2 San Pedro Mines	23.74174	−99.14599	X	X	
AMNH	84798	F	*Sanguinolenta*	24/04/1888	Mexico: Tamaulipas, Victoria	23.74174	−99.14599		X	
AMNH	84799	M	*Sanguinolenta*	16/04/1888	Mexico: Tamaulipas, Victoria	23.74174	−99.14599	X	X	
AMNH	84802	F	*Sanguinolenta*	07/06/1889	Mexico: Nuevo Leon, Camp 3 Boquillo	25.364444	−100.405278	X	X	
AMNH	84803	F	*Sanguinolenta*	17/06/1889	Mexico: Nuevo Leon, Boque Nagro	25.364444	−100.405278	X	X	
AMNH	84805	F	*Sanguinolenta*	ND	Mexico: Verzacruz, Jalapa	19.53124	−96.91589		X	
AMNH	91849	M	*Bidentata*	26/04/1904	Mexico: Sinaloa, Juan Lisiarraga Mt.	23.030053	−105.435033	X	X	
AMNH	91851	M	*Bidentata*	27/04/1904	Mexico: Sinaloa, Juan Lisiarraga Mt.	23.030053	−105.435033	X	X	
AMNH	91853	M	*Bidentata*	27/04/1904	Mexico: Sinaloa, Juan Lisiarraga Mt.	23.030053	−105.435033	X		
AMNH	91856	F	*Bidentata*	27/04/1904	Mexico: Sinaloa, Juan Lisiarraga Mt.	23.030053	−105.435033	X	X	
AMNH	91857	F	*Bidentata*	27/04/1904	Mexico: Sinaloa, Juan Lisiarraga Mt.	23.030053	−105.435033	X	X	
AMNH	102362	M	*Citrea*	??/12/1905	Costa Rica: San Jose	9.9333	−84.0833	X	X	
AMNH	102363	M	*Citrea*	15/12/1906	Costa Rica: Escazu	9.91887	−84.13989		X	
AMNH	105036	M	*Bidentata*	18/02/1905	Mexico: Nayarit, Tepic, Ojo de Agua nr. Amatlan de Cañas	20.80469	−104.421967	X	X	
AMNH	105037	M	*Bidentata*	15/02/1905	Mexico: Nayarit, Amatlan de Cañas	20.80469	−104.421967	X	X	
AMNH	105038	M	*Bidentata*	26/04/1905	Mexico: Jalisco, Wakenakili Mountains	21.8	−103.866667	X	X	
AMNH	105039	M	*Bidentata*	26/04/1905	Mexico: Jalisco, Wakenakili Mountains	21.8	−103.866667	X	X	
AMNH	105040	M	*Bidentata*	25/04/1906	Mexico: Jalisco, Wakenakili Mountains	21.8	−103.866667	X	X	
AMNH	105041	M	*Bidentata*	26/04/1905	Mexico: Jalisco, Wakenakili Mountains	21.8	−103.866667	X	X	
AMNH	105042	M	*Bidentata*	26/04/1905	Mexico: Jalisco, Wakenakili Mountains	21.8	−103.866667	X	X	
AMNH	105043	F	*Bidentata*	15/02/1905	Mexico: Nayarit, Amatlan de Cañas	20.80469	−104.421967		X	
AMNH	105044	F	*Bidentata*	24/04/1906	Mexico: Jalisco, Wakenakili Mountains	21.8	−103.866667	X	X	
AMNH	105046	F	*Bidentata*	25/04/1906	Mexico: Jalisco, Wakenakili Mountains	21.8	−103.866667	X	X	
AMNH	106860	F	*Citrea*	26/08/1901	Panama: Chiriqui Province, Boquete	8.78024	−82.44136		X	
AMNH	106861	F	*Citrea*	05/09/1901	Panama: Chiriqui Province, Boquete	8.78024	−82.44136	X	X	
AMNH	106862	F	*Citrea*	08/09/1901	Panama: Chiriqui Province, Boquete	8.78024	−82.44136	X	X	
AMNH	106863	F	*Citrea*	06/09/1901	Panama: Chiriqui Province, Boquete	8.78024	−82.44136	X	X	
AMNH	106864	F	*Citrea*	04/09/1901	Panama: Chiriqui Province, Boquete	8.78024	−82.44136	X	X	
AMNH	123723	M	*Citrea*	ND	Costa Rica: Irazu	9.983	−83.85	X	X	
AMNH	144703	M	*Sanguinolenta*	11/04/1917	Guatemala: near Jinotega	13.1	-86	X	X	
AMNH	153426	M	*Sanguinolenta*	29/03/1897	Mexico: Verzacruz, Jalapa	19.53124	−96.91589	X	X	
AMNH	153427	M	*Sanguinolenta*	05/04/1897	Mexico: Verzacruz, Jalapa	19.53124	−96.91589	X	X	
AMNH	153428	F	*Sanguinolenta*	29/03/1897	Mexico: Verzacruz, Jalapa	19.53124	−96.91589	X	X	
AMNH	388795	M	*Sanguinolenta*	22/04/1952	Mexico: San Luis Potosi, El Lobo, above Xintitlar	21.41	−99.02	X	X	
AMNH	392573	M	*Citrea*	04/05/1920	Costa Rica: Irazu Volcano	9.983	−83.85	X	X	
AMNH	392574	M	*Citrea*	05/05/1920	Costa Rica: Irazu Volcano	9.983	−83.85	X	X	
AMNH	392578	F	*Citrea*	13/05/1920	Costa Rica: Irazu Volcano	9.983	−83.85	X	X	
AMNH	392579	M	*Citrea*	27/051920	Costa Rica: Aqua Caliente	10.35	−85.067	X	X	
AMNH	392580	M	*Citrea*	09/06/1920	Costa Rica: Aqua Caliente	10.35	−85.067	X	X	
AMNH	392581	M	*Citrea*	11/06/1920	Costa Rica: Aqua Caliente	10.35	−85.067	X	X	
AMNH	392582	F	*Citrea*	20/05/1920	Costa Rica: Aqua Caliente	10.35	−85.067	X	X	
AMNH	392583	F	*Citrea*	11/06/1920	Costa Rica: Aqua Caliente	10.35	−85.067	X	X	
AMNH	392584	M	*Citrea*	12/07/1920	Costa Rica: Navarrito	9.808752	−83.877565	X	X	
AMNH	392585	M	*Citrea*	17/05/1925	Costa Rica: Cartago	9.867	−83.917	X	X	
AMNH	392586	M	*Citrea*	??/??/1924	Costa Rica: San Jeronimo	9.856615	−83.7937	X	X	
AMNH	398430	M	*Sanguinolenta*	06/08/1924	Guatemala: Finca	13.997	−90.676	X	X	
AMNH	398432	M	*Sanguinolenta*	02/04/1924	Guatemala: La Perla	15.614	−91.112	X	X	
AMNH	398433	F	*Sanguinolenta*	02/04/1924	Guatemala: La Perla	15.614	−91.112	X	X	
AMNH	398436	F	*Sanguinolenta*	30/06/1924	Guatemala: San Lucas	14.583333	−91.186111	X	X	
AMNH	398437	M	*Sanguinolenta*	09/06/1928	Guatemala: San Lucas	14.583333	−91.186111	X	X	
AMNH	398438	M	*Sanguinolenta*	14/06/1928	Guatemala: San Lucas	14.583333	−91.186111	X	X	
AMNH	398439	M	*Sanguinolenta*	02/03/1925	Guatemala: Finca El Soche	15.383	−90.833	X	X	
AMNH	398440	M	*Sanguinolenta*	10/02/1925	Guatemala: Finca La Primavera	15.47083	−90.3708267	X	X	
AMNH	398441	M	*Sanguinolenta*	05/03/1927	Guatemala: Barrillos	15.804	−91.316	X	X	
AMNH	406784	M	*Bidentata*	10/02/1908	Mexico: Morelos, Cuernavaca	18.9242095	−99.2215659	X	X	
AMNH	406787	F	*Bidentata*	16/02/1908	Mexico: Morelos, Cuernavaca	18.9242095	−99.2215659	X	X	
AMNH	406788	M	*Bidentata*	18/05/1909	Mexico: Nayarit, San Blas	21.5413	−105.2847	X	X	
AMNH	441314	M	*Sanguinolenta*	26/04/1888	Mexico: Tamaulipas, Victoria	23.74174	−99.14599	X	X	
AMNH	510303	M	*Bidentata*	03/04/1891	Mexico: Nayarit, Sierra de Alica	21.70095	−104.754695	X	X	
AMNH	510304	M	*Bidentata*	05/04/1891	Mexico: Nayarit, Sierra de Alica	21.70095	−104.754695	X	X	
AMNH	510305	M	*Bidentata*	22/04/1891	Mexico: Nayarit, Sierra de Alica	21.70095	−104.754695	X	X	
AMNH	510306	M	*Bidentata*	05/01/1892	Mexico: Jalisco, Barranca del Mesquitan, Guadalajara	20.652119	−103.384799	X	X	
AMNH	510307	F	*Bidentata*	17/01/1892	Mexico: Jalisco, Barranca del Portillo, Guadalajara	20.652119	−103.384799	X	X	
AMNH	510308	F	*Bidentata*	14/12/1891	Mexico: Nayarit, Barranca del Oro, Tepic	20.933	−104.483	X	X	
AMNH	510309	M	*Flammea*	04/05/1897	Mexico: Nayarit, Maria Madre Island	21.7402778	−106.654722	X	X	
AMNH	510310	M	*Flammea*	07/05/1897	Mexico: Nayarit, Maria Madre Island	21.7402778	−106.654722	X	X	
AMNH	510311	M	*Flammea*	11/05/1897	Mexico: Nayarit, Maria Madre Island	21.7402778	−106.654722	X	X	
AMNH	510312	M	*Flammea*	09/05/1897	Mexico: Nayarit, Maria Madre Island	21.7402778	−106.654722	X	X	
AMNH	510315	F	*Flammea*	05/05/1897	Mexico: Nayarit, Maria Madre Island	21.7402778	−106.654722	X	X	
AMNH	510316	F	*Flammea*	06/05/1897	Mexico: Nayarit, Maria Madre Island	21.7402778	−106.654722	X	X	
AMNH	510317	F	*Flammea*	09/05/1897	Mexico: Nayarit, Maria Madre Island	21.7402778	−106.654722	X	X	
AMNH	510318	F	*Citrea*	ND	Costa Rica: San Jose?	9.9333	−84.0833	X	X	
AMNH	510320	M	*Citrea*	ND	Costa Rica: San Jose?	9.9333	−84.0833	X	X	
AMNH	510321	M	*Citrea*	??/??/1864	Costa Rica: Cartago	9.867	−83.917	X	X	
AMNH	510322	M	*Citrea*	08/06/1894	Costa Rica: La Isla Braza (Brava?)	10.710627	−83.693772	X	X	
AMNH	510323	M	*Citrea*	02/11/1897	Costa Rica: San José	9.9333	−84.0833	X	X	
AMNH	510324	F	*Citrea*	05/12/1890	Costa Rica: San José	9.9333	−84.0833	X	X	
AMNH	510325	F	*Citrea*	30/04/1893	Costa Rica: San José	9.9333	−84.0833	X	X	
AMNH	510326	M	*Sanguinolenta*	ND	Mexico: Veracruz, Orizaba	18.8505	−97.1036	X	X	
AMNH	510327	M	*Sanguinolenta*	ND	Guatemala: Vera Paz	15.499998	−90.333332	X	X	
AMNH	510328	F	*Citrea*	10/02/????	Panama: Chiriqui Province, Boquete	8.78024	−82.44136	X	X	
AMNH	510329	F	*Citrea*	ND	Panama: Chiriqui Province, Veraqua	8.1000004	−80.9833298	X	X	
AMNH	510330	F	*Citrea*	ND	Panama: Chiriqui Province, Veraqua	8.1000004	−80.9833298		X	
AMNH	510331	M	*Citrea*	??/??/1900	Panama: Chiriqui Province	8.78024	−82.44136	X	X	
AMNH	510332	M	*Citrea*	09/04/1905	Panama: Chiriqui Province, Boquete	8.78024	−82.44136	X	X	
AMNH	510333	M	*Citrea*	07/03/1905	Panama: Chiriqui Province, Boquete	8.78024	−82.44136	X	X	
AMNH	510336	F	*Citrea*	27/01/1902	Panama: Reported as ”Brava Island” but most likely brom the vicinity of Boquete.	8.78024	−82.44136	X	X	
AMNH	510337	F	*Citrea*	27/01/1902	Panama: Reported as ”Brava Island” but most likely brom the vicinity of Boquete.	8.78024	−82.44136	X	X	
AMNH	510339	F	*Citrea*	29/01/1902	Panama: Reported as ”Brava Island” but most likely brom the vicinity of Boquete.	8.78024	−82.44136		X	
AMNH	510340	F	*Citrea*	15/01/1902	Panama: Reported as ”Jicaron Island” but most likely brom the vicinity of Boquete.	8.78024	−82.44136		X	
AMNH	648707	F	*Sanguinolenta*	10/05/1953	Mexico: Tamaulipas, Rancho del Cielo, 5 mi. N.W. Gomez Favias	23.274725	−99.276218	X	X	
AMNH	784663	M	*Sanguinolenta*	ND	Mexico: Chiapas, El Triunfo	15.666073	−92.800064	X	X	
AMNH	806451	M	*Bidentata*	11/02/1908	Mexico: Morelos, Cuernavaca	18.9242095	−99.2215659	X	X	
AMNH	183004	M	*Citrea*	05/03/1924	Panama: Chiriqui Province, Cerro Flores	8.4	−82.317	X	X	
AMNH	183019	F	*Citrea*	07/03/1924	Panama: Chiriqui Province, Cerro Flores	8.4	−82.317	X	X	
AMNH	183017	F	*Citrea*	05/03/1924	Panama: Chiriqui Province, Cerro Flores	8.4	−82.317	X	X	
AMNH	183015	M	*Citrea*	11/03/1924	Panama: Chiriqui Province, Cerro Flores	8.4	−82.317	X	X	
AMNH	183014	M	*Citrea*	11/03/1924	Panama: Chiriqui Province, Cerro Flores	8.4	−82.317	X	X	
AMNH	183011	M	*Citrea*	08/03/1924	Panama: Chiriqui Province, Cerro Flores	8.4	−82.317	X	X	
AMNH	183009	M	*Citrea*	07/03/1924	Panama: Chiriqui Province, Cerro Flores	8.4	−82.317	X	X	
AMNH	183006	M	*Citrea*	06/03/1924	Panama: Chiriqui Province, Cerro Flores	8.4	−82.317	X	X	
MVZ	187251	F	*Sanguinolenta*	11/01/2012	Guatemala: Quetzaltenango, Volcan Lacandon, Municipio Colomba Costa Cuca	14.81	−91.74			X
MVZ	188292	M	*Sanguinolenta*	24/06/2012	Mexico: Chiapas, Cerro Boqueron, Mpio. Motozintla	15.23	92.3			X
KU	B-27251	ND	*Citrea*	14/03/2016	Costa Rica: San Jose Province	ND	ND			X
KU	B-46479	ND	*Citrea*	14/04/2016	Panama: Chiriqui Province	ND	ND			X
LSU	SLA 358	F	*Sanguinolenta*	03/03/2004	El Salvador: Chalatenango, La Laguna, La Montanona	14.13	88.91			X

We assembled the resulting reads into a SNP library using ipyrad (Eaton & Overcast, 2020) using the reference approach and mapping our reads to the Northern Cardinal (*Cardinalis cardinalis*) reference genome (Sin, Lu & Edwards, 2020, GenBank SRA BioProject accession PRJNA642398). We filtered reads for quality by trimming reads with a PHRED score of less than 43 from the 3′ end. Since many biological processes can affect the level of genetic differentiation between two samples, the clustering threshold at which two sequences are called as orthologous cannot be assumed by default, and must be optimized empirically for each study system (McCartney-Melstad, Gidiş & Shaffer, 2019). We selected a clustering threshold of 88% based on four metrics: (1) Pearson correlation coefficient between pairwise genetic distance and percentage of missing data, (2) cumulative variance explained by the first three Principal Components, (3) total SNPs recovered, and (4) total loci recovered (McCartney-Melstad, Gidiş & Shaffer, 2019). For the final alignment, we retained loci that were present in 75% of the samples. For analyses that require unlinked loci, we further filtered our dataset using the R packages *SNPfiltR* (DeRaad, 2021) and *vcfR* (Knaus & Grünwald, 2017), retaining only loci that were more than 1000bp away from one another.

### Color data

We measured the color of seven feather patches (Crown, nape, tail throat, breast, upper belly and lower belly; shown in Fig. 1C) of 109 study skins (Table 1) held at the AMNH Ornithology Collection and Museo de Zoología de la Facultad de Ciencias (MZFC), using an OceanOptics USB 2000+ spectrophotometer paired with a pulsed xenon light source. Six of these patches are carotenoid-colored, and one (tail) is melanin-colored. The measuring probe was directed perpendicularly to the feather surface and ambient light was blocked by a drilled rubber stopper surrounding the tip of the probe. We obtained spectrographic measurements of feather reflectance in the avian visual range of 300-700 nm. Feather patch reflectance was measured relative to the reflectance of a WS-2 white standard (OceanOptics) and then processed in the R package *pavo* (Maia et al., 2019) to smooth the spectrographic curves and correct for electric noise. From the smoothed and noise-corrected spectrographic data of each feather patch we obtained three standard variables, hue (H3), chroma (S8) and absolute brightness (B1). We used H3 instead of the more commonly used H1 because the spectral curves of carotenoid coloration show a distinct “shoulder” shape, always having its peak (*R*_max_) at 700nm, showing no variation and rendering the variable uninformative. We found that calculating the peak intensity at *R*_mid_ instead of *R*_max_ (H3) better reflects the observed variation in carotenoid coloration. In addition, we also obtained the red chroma (SR1) of each patch, calculated as the proportion of the area under the curve in the red region (between 580 nm and 700 nm), as a complementary measure of both hue and red spectral purity. We followed the same procedure for the tail patch, which shows melanin-based coloration, to make results comparable between feather patches. We modeled the visual response to the measured color under the noise-limited visual receptor model described by Vorobyev & Osorio (1998) as implemented in the R package *pavo* (Maia et al., 2019) to test if any observed differences in color translate into perceptual differences in the avian visual system. The visual model we used to simulate the quantum catch was that of the Blue Tit (*Cyanistes caeruleus*), as it is the phylogenetically closest to our study species from models available in the package. The source of illumination we chose was sunlight filtered through forest canopy, as it most accurately reflects the light conditions our study species is likely to be seen under in the wild.

### Morphometric data

We examined 141 study skins of *Piranga bidentata,* held at the American Museum of Natural History (AMNH) and the Museo de Zoología de la Facultad de Ciencias (MZFC) zoological collections, covering all four-described subspecies and the majority of the species’ geographical distribution (Table 1). Since juvenile males have plumage that strongly resembles that of females, we removed from analysis any specimen with yellow plumage that lacked age data that identified it as an adult (*e.g.*, skull ossification, presence of bursa or notes on gonad development). This also allows us to mitigate the error caused by allometric changes during different stages of individual development. We used a Mitutoyo digital caliper to take external morphological measurements of standard body parts. To reduce measurement error, we used the average of triplicate measurements of each body part for analysis. We discarded the measurements of tail length because we found it difficult to replicate and highly affected by terminal wear on feathers.

### Climate data

We used the R package *raster* (Hijmans, 2019) to extract the 19 climate variables from the CHELSA 2.1 climatology data set (Karger et al., 2017; Karger et al., 2021) in a circular 1km buffer around the geographic coordinates of each voucher specimen. For population-level analyses, we calculated a convex hull containing all specimens belonging to that population and obtained the climate variables around the hull centroid. These climatic variables correspond roughly to temperature, precipitation and seasonality variables, and many are highly correlated. To reduce model overfitting and mitigate the risk of multicollinearity, we avoided using variables with more than |0.7| correlation in the same model as suggested by Dormann et al. (2013). To test whether there was a correlation between feather color and vegetation cover, we obtained an estimate the percentage tree cover from the Moderate Resolution Imaging Spectroradiometer (ModIS) satellite though their MOD44B.006 Terra Vegetation Continuous Fields Yearly 250 m dataset (DiMiceli et al., 2015), accessed through the Google Earth Engine portal (Gorelick et al., 2017) and operated by NASA. We calculated the median vegetation cover from 19 yearly images spanning the period from 2000 to 2018. Finally, we calculated pairwise geographic distance between each collection point and between population centroids using the great circle distance algorithm implemented in the R package *geodist* (Padgham & Sunmer, 2019).

### Analysis

**Figure 2:**
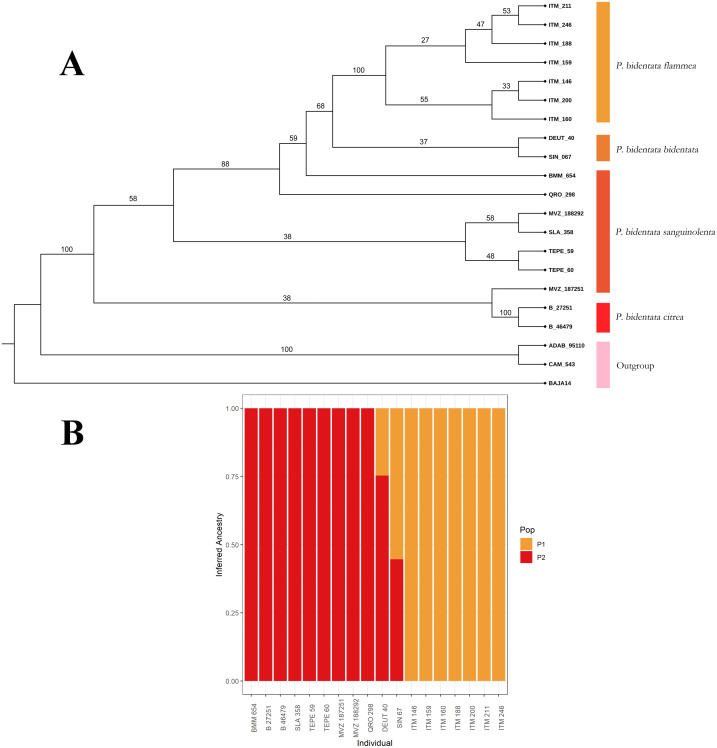
Coalescent tree. (A) Coalescent tree of 81,739 SNPs. We recover the whole of *Piranga bidentata* as a monophyletic group. Of the described subspecies, we only recovered *P.b. flammea* and *P.b. citrea* as monophyletic groups, although we find evidence of clustering with respect of two major color groups. The outgroup is composed of two *Piranga roseogularis* samples (ADAB 95110 and CAM 543), as well as one *Cardinalis cardinalis* sample (BAJA14) (B) ADMIXTURE bar chart showing the inferred ancestry of each individual in our sample, with a *K* value of 2. The clusters we observe correspond to the two color morph groups, with our two individuals from the *bidentata* subspecies appearing as having mixed ancestry.

To first assess if genetic and color variation are phylogenetically structured (color groups corresponding to described subspecies), we constructed a phylogenetic tree with our SNPs data. We used the SVDQuartets (Chifman & Kubatko, 2014) algorithm implemented in PAUP* (Swofford, 2002) to obtain a coalescent tree topology. We generated 100 bootstrap replicates to assess branch support in the resulting consensus phylogenetic reconstruction, which we present in Fig. 2. We then assessed visually if members of the described subspecies clustered together into monophyletic groups. We also assessed these groups using the Bayesian clustering algorithm implemented in the software ADMIXTURE (Alexander, Novembre & Lange, 2009), using a *K* determined by minimizing cross-validation error. This allowed us to estimate the ancestry of individuals within our sample.

As the study species is obviously dichromatic, we performed separate analysis on colorimetric data by sex. We explored variation in color by performing one-way analysis of variance (ANOVA) on the calculated color variables (B1, S8, H3 and S1R) with the subspecies as a factor. The results of this analysis are in the Tables S1 and S2. To explore if intraspecific variation is consistent with Gloger’s Rule we constructed linear models with plumage brightness (B1) as a response variable and mean annual precipitation (*bio12*) and mean annual temperature (*bio1*) as predictors. Ventral coloration is under stronger sexual selection compared to dorsal coloration in many bird taxa (Shultz & Burns, 2017; Marcondes & Brumfield, 2019). Since ventral color is relatively uniform patterned in this species, we took the average of the four ventral patches as representative of overall carotenoid color in subsequent analyses.

To further assess if genetic clusters match color groups, we performed linear discriminant analyses on ventral coloration variables on females (using plumage brightness, hue and chroma; B1, H3 and S8) and males (using plumage brightness, hue, overall chroma and red chroma; B1, H3, S8 and S1R) separately.

We followed a similar procedure for morphometric data, using one-way ANOVA to test for differences in each measured variable between subspecies (Table S3). Since one morphological variable was one order of magnitude larger than the rest, we rescaled all morphological data by *log*-converting it. To test if there were differences in body size among the described subspecies, we carried out an analysis of variance (ANOVA) with subspecies as a factor for each of the variables we measured. The results of these tests are shown in Table S3. To condense this morphological variation, we carried out a principal component analysis (PCA) on our morphological data matrix and obtained a set of principal components, the first of which explains 79.4% of the total variation, and is positively correlated almost entirely with wing chord. The second principal component explains 12.76% of variance and is positively correlated with the rest of the morphological variables. To test if this species follows the pattern known as Bergmann’s Rule, we constructed a linear regression model with the first PCA score value as a response variable and mean annual temperature (*bio1*) as the predictor.

To distinguish between the effect of genetic distance, geographic distance, and environmental variables, we used multiple regression on matrices (MRM) as implemented in the R package *ecodist* (Goslee & Urban, 2007), which tests for relationships between two or more distance matrices using matrix permutation. We also tested the relative effects of geographic distance and rainfall divergence on allele frequencies using a Bayesian MCMC framework implemented in the R package BEDASSLE (Bradburd, Ralph & Coop, 2013). We ran BEDASSLE for 1,000,000 generations using the beta binomial model, which allows populations to diverge from model expectations due to overdispersion. We used a matrix of Euclidean distances on rainfall, calculated from the CHELSA climatology data set (Karger et al., 2021). To test for the effects of selection, we used Bayescan 2.1 (Foll & Gaggiotti, 2008) to identify loci under selection in the color morphs. These tests allowed us to test the likelihood of the null model of isolation by distance (IBD), divergence due to drift, and local adaptation. Since we lack a one-to-one match between genomic sequences and color data, we carried out this analysis using the average spectrum per subspecies and an *Fst* distance matrix between the four subspecies.

## Results

### Genomics

From our data set of 84,739 SNP loci we obtained a coalescent tree topology (shown in Fig. 2A) which grouped all of the *Piranga bidentata* samples into a single well-supported clade, supporting the monophyly of *Piranga bidentata*. The two western groups with orange male plumage (*bidentata* and *flammea*) cluster into one subclade with middling support. Samples from populations with red male plumage cluster together, although the topology doesn’t reflect the geographic structure of the samples, and we did not recover the subspecies *P. b. sanguinolenta* as monophyletic. Despite our limited sampling, we found some evidence for genetic structure in this species.

Our ADMIXTURE results (*K* = 2, Fig. 2B) show that most of our samples cluster unambiguously into two groups. These groups correspond to the orange color morph (subspecies *bidentata* and *flammea*) and the red color morph (subspecies *citrea* and *sanguinolenta*). Our two samples from the *bidentata* subspecies appear in the analysis as admixed between these two groups.

We performed two Bayescan runs of 100,000 generations each on a subset of 8,440 unlinked loci, one with prior neutral model odds of 100 and the other with odds of 1,000. In both runs we failed to detect any loci as statistically significant outliers in *Fst*, suggesting the markers in our data set are selectively neutral. However, this method risks low statistical power with small sample sizes, so these results should be interpreted carefully. A more extensive sampling is needed to fully assess selection in this taxon.

Our BEDASSLE run resulted in an aE/aD posterior distribution with a mean of 15,091 which suggests that the effect of rainfall on allele frequencies is relatively small compared to the effect of geographic distance (A difference of one mm of annual rainfall having a comparable effect as about 15km of lateral movement).

### Color

The ANOVA of the color data (Table S1) showed a break in males’ carotenoid coloration for all four of the variables we computed (H3, S8, SR1 and B1), with males of the orange group (subspecies *bidentata* and *flammea*) having brighter, more orange and less spectrally saturated plumage color than males from the red group (subspecies *sanguinolenta* and *citrea*).

**Figure 3:**
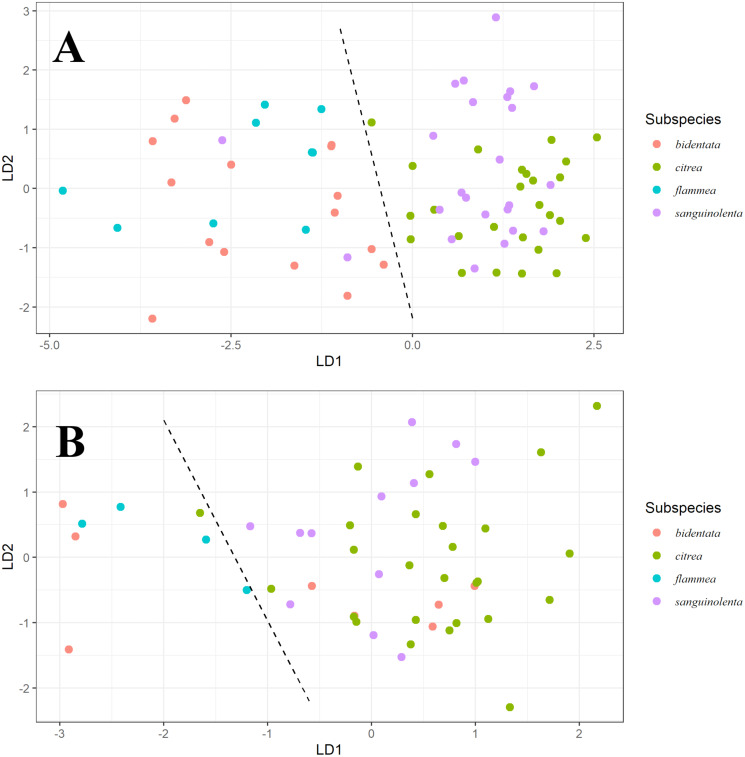
Linear discriminant analysis for males and females. Linear Discriminant Analysis on ventral color for males (A) and females (B). Linear discriminants were calculated from the variables B1, H3 and S8 for both, with the addition of S1R for males. For males, we can observe discrimination between the orange (*bidentata* and *flammea*) and red (*citrea* and *sanguinolenta*) groups, but not between the subspecies comprising each group. For females, we can see a similar pattern, but less strongly differentiated.

Our LDA result shows that for males (Fig. 3A), ventral color variables B1, H3, S8 and S1R distinguish two color groups congruent with the genetic clusters we detected. However, these predictors can’t distinguish between the described subspecies that form each of the groups. We found a similar pattern for females (Fig. 3B), although the separation is much less clear.

**Figure 4:**
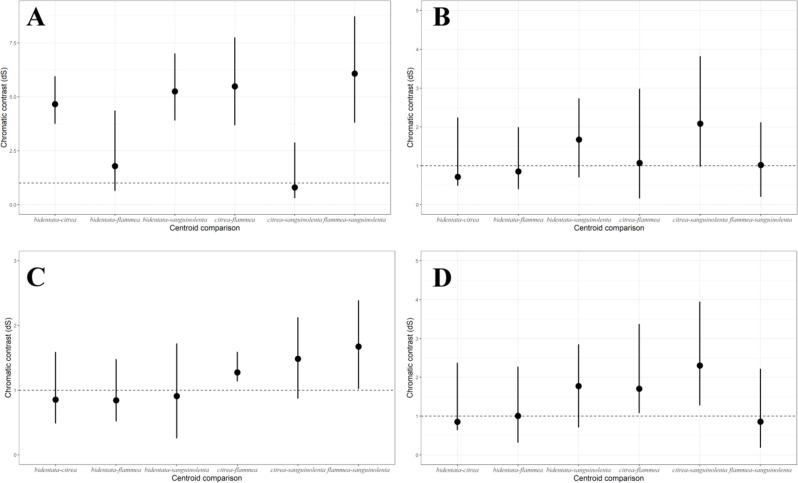
Pairwise comparisons of plumage color under the noise-constrained receptor model. (A) Male ventral carotenoid coloration (Throat, breast, upper and lower belly); (B) female ventral carotenoid coloration (Throat, breast, upper and lower belly); (C) male melanin coloration (Tail); (D) female melanin coloration (Tail). (A) shows more variation between the two colors groups than among the color groups. (B, C & D) show similar patterns and magnitudes of variation.

Under the noise-constrained visual receptor modeling (Vorobyev & Osorio, 1998), the average difference in color that we measured between the red and orange groups is higher than the perceptual threshold (*i.e.,* larger than 1 JND, for *just-noticeable difference* Fig. 4A), which indicates that the colors are sufficiently different that birds are capable of discriminating between them. Notably, there was also a slight but noticeable and statistically significant difference between the West mainland and the Tres Marias orange birds, with the island birds having brighter orange plumage. The pairwise differences in ventral color between subspecies of the same color group (*bidentata*-*flammea* and *sanguinolenta*-*citrea*) are much smaller.

Females were generally consistent in their ventral carotenoid coloration, showing no significant variation in either hue or chroma among subspecies (Table S2). We found a small but statistically significant variation in plumage brightness among subspecies, which follows the same overall pattern as in the males. Although the pairwise differences in overall carotenoid color between subspecies are larger than one 1 JND, the threshold falls within the confidence interval (Fig. 4B).

For melanin coloration, we found a similar pattern and magnitude of divergence between subspecies in both males (Fig. 4C) and females (Fig. 4D).

**Figure 5:**
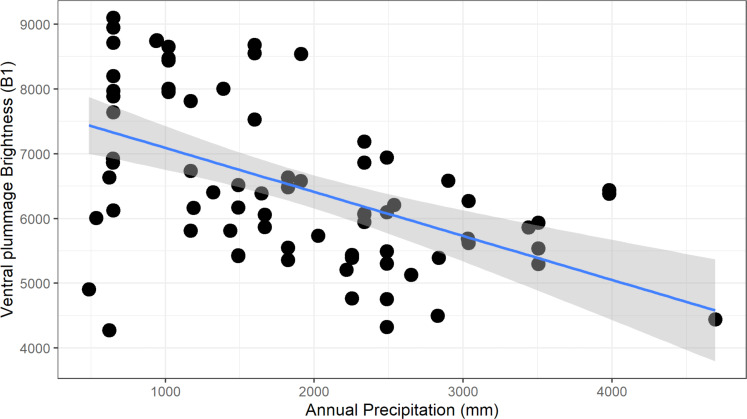
Negative relationship between ventral plumage brightness and annual precipitation for males. This relationship is consistent with the ecogeographic pattern known as Gloger’s rule, which predicts that organisms living in more humid climates will tend to be darker.

For males, we found a significant negative relationship between plumage brightness (B1) and annual precipitation (*bio12*), for both carotenoid (ventral, *F*_1–73_ = 26.6, adj. *R*^2^ = 0.26, *p* > 0.001, Fig. 5) and melanin (tail, *F*_1–73_ = 13.62, adj. *R*^2^ = 0.15, *p* < 0.001) feather patches. We found a similar negative trend in female color, although the coefficients of determination were lower (ventral, *F*_1–48_ = 12.62, adj.*R*^2^ = 0.19, *p* < 0.001; tail, *F*_1–47_ = 6.34, adj. *R*^2^ = 0.10, *p* = 0.015). These results show that this species follows the ecogeographical pattern known as Gloger’s Rule, which predicts that among ectothermic species, individuals found in habitats with higher humidity will tend to have darker coloration.

We found a similar negative relationship between ventral plumage brightness and isothermality *bio3* (males, *F*_1–73_ = 27.42, adj. *R*^2^ = 0.26, *p* > 0.001; females, *F*_1–48_ = 8.76, adj. *R*^2^ = 0.14, *p* = 0.005), as well as a statistically significant but weak negative relation between tail brightness and isothermality (*F*_1–73_ = 7.22, adj. *R*^2^ = 0.08, *p* = 0.009) in males.

To further test whether there was support for the hypothesis that Gloger’s Rule is at least partially due to selection of darker individuals in habitats with more dense vegetation, we tested for a relationship between plumage brightness and remotely sensed vegetation coverage calculated from ModIS measurements. We found a significant relationship between plumage brightness (*F*_1–60_ = 16.55, adj. *R*^2^ = 0.2, *p* < 0.001) and vegetation cover, but not for red chroma (*F*_1–60_ = 1.37, adj. *R*^2^ < 0.001, *p* = 0.246) and vegetation coverage, as calculated from ModIS measurements. The results of MRM analysis showed that for males there is statistically significant positive relationship between differences in breast feather color and geographic distance (*F* = 73.26, *R*^2^ = 0.037, *p* = 0.001), and no significant relationship between breast color differences and genetic distance (*F* = 1.97, *R*^2^ = 0.33, *p* = 0.38) or difference in vegetation cover (*F* = 9.26, *R*^2^ = 0.004, *p* = 0.26). However, we found a significant positive relationship between differences in mean annual rainfall and differences in breast color (*F* = 439.229, *R*^2^ = 0.19, *p* = 0.001). For females, we found no relationship between color distance and geographic distance (*F* = 16.064, *R*^2^ = 0.015, *p* = 0.128) or differences in rainfall (*F* = 1.92, *R*^2^ = 0.002, *p* = 0.544).

The MRM model with male breast color distance as response variable and *Fst*, geographic distance and rainfall distance matrices as joint predictors shows a not statistically significant but very large proportion of color variance is explained by these three factors (*F* = 26.39, *R*^2^ = 0.975, *p* = 0.07). We obtained a similar result with female breast color distance, although the trend is much weaker (*F* = 0.50, *R*^2^ = 0.43, *p* = 0.79)

### Morphometrics

We found no significant sexual dimorphism in this group for five of the six morphological variables we measured. The only measurement for which sexual dimorphism was statistically significant was the wing chord. Of the six measurements, we found significant variation between subspecies in 5 of them (Table S3). The first two Principal Components resulting from our PCA explained 73.48% of the observed variation. Principal Component 1 was strongly positively correlated with wing chord, and Principal Component 2 was correlated with the other morphological variables we measured, and thus serves as a proxy for overall body size. We found that PC1 scores separate the subspecies *bidentata* from the rest, while PC2 separates the Mexican subspecies (*bidentata* and *sanguinolenta*) from the remaining two. However, all subspecies showed a high degree of overlap in morphometrics (See Supplemental Information). MRM results showed a small but significant relationship between our proxy for body size (PC2) and geographic distance (*F* = 202.57, *R*^2^ = 0.02, *p* = 0.001) but not between PC2 and mean annual temperature (*F* = 0.3, *R*^2^ = 0.0003, *p* = 0.84).

## Discussion

Our results show that there is significant variation in male feather coloration and body size among populations of *Piranga bidentata*, as well as evidence of genetic structure. This variation in color covaries in different amounts with genetic structure, geographic distance and climatic variables. These results suggest support for the hypothesis that the observed variation in color is partially the result of local adaptation, phenotypic plasticity or a combination of the two. The most striking of these differences is the change in male carotenoid coloration from orange in the Western Mexico subspecies (*bidentata* and *flammea*) to red in the Eastern Mexico and Central American subspecies (*citrea* and *sanguinolenta*), which we found to be related to mean annual rainfall. Overall plumage brightness also differs among groups, and this variation is related to mean annual rainfall, but not to vegetation coverage calculated from ModIS measurements. These results are consistent with the well-known ecogeographical pattern known as Gloger’s rule (Gloger, 1833). For body size, we found no relationship between a measure of body size and mean annual temperature, but there is a significant relationship between body size and geographic distance, suggesting that the mechanism at work is isolation by distance. A genomic scan for loci associated with body size could clarify the role of genetic differentiation on variation of body size.

### Plumage color

Plumage carotenoids are obtained exclusively from the diet, but the relationship between carotenoid ingestion and plumage color expression is not necessarily direct. The deposition of red and orange carotenoids (*e.g.*, canthaxanthin and astaxanthin) requires metabolic conversion through ketolation of dietary yellow carotenoids (*e.g.*, zeaxanthin). Although the metabolic pathway is not fully understood, evidence from several studies suggests that CYP2J19, a member of the P450 cytochrome gene family, is implicated in the ketolation reaction of dietary carotenoids (Mundy et al., 2016; Lopes et al., 2016; Emerling, 2018; Sin, Lu & Edwards, 2020). The enzymatic activity of CYP2J19 and other cytochrome proteins appears to be highly correlated with overall mitochondrial activity (Hill et al., 2019; Cantarero et al., 2020), which potentially maintains the honesty of modified carotenoid ornamentation as a signal of individual quality. While Koch et al. (2019) found that the evidence linking carotenoid deposition to several measures of individual performance is weak, other studies show evidence that carotenoid pigmentation is correlated with testosterone levels (Khalil et al., 2020).

While the exact chemical makeup of the feather carotenoids in this species has not yet been studied, closely related species of the genus *Piranga* derive their yellow feather coloration from dietary zeaxanthin and their red and orange coloration from a combination of several 4-keto-carotenoids such as canthaxanthin and astaxanthin (Hudon, 1991; Lopes et al., 2016). This seems to be largely conserved in the genus, with a few exceptions (Hudon, 1991). Since the difference in color that we observed appears to be continuously variable, it is likely that observed differences in color result from changes in the relative proportions of several carotenoids being deposited in feathers. This ratio is likely associated to some degree with the activity of CYP2J19-like gene products, which perform these ketolation reactions.

Another potential source of variation in coloration is the effect of differential sexual selection between populations. Our pairwise comparisons (Fig. 3) show similar patterns and magnitude of variation between subspecies for female carotenoid (3B), male melanin (3C) and female melanin (3D), but much larger differences in male ventral carotenoid colors (3A). As ventral coloration has been found to be under heightened sexual selection compared to other plumage patches (Shultz & Burns, 2017), this greater variation could be the result of stronger sexual selection for ketocarotenoid coloration in the red group (*sanguinolenta* and *citrea*). A series of studies on foraging behavior and characterization of dietary carotenoids available to this species would help illuminate other possible mechanisms modulating color expression.

### Gloger’s rule

We found that in *Piranga bidentata*, darker and redder coloration in males is correlated with higher environmental humidity (measured as annual rainfall), compared to orange coloration. This is consistent with the ecogeographical pattern known as Gloger’s rule, which in its original formulation predicts that for endothermic organisms, darker coloration is found in hotter and more humid environments (Gloger, 1833). This pattern is widely observed in birds and mammals, and a study by Zink & Remsen (1986). found that a majority of North American bird species studied conform to it to some degree.

One of the most widely accepted hypotheses to explain Gloger’s rule is selection for darker and more cryptic plumage in environments with dense vegetation and thus lower environmental light levels (McNaught & Owens, 2002). Alternative explanations involving other, non-mutually exclusive processes have also been proposed. These include increased feather resistance to mechanical wear (Bonser, 1995), increased resistance to bacterial and fungal degradation of feathers (Burtt Jr & Ichida, 2004; Gunderson et al., 2008), and pleiotropic effects from selection on other traits which increase with humidity such as immune responsiveness and overall mitochondrial activity (Johnson & Hill 2013; Hill et al., 2019; Cantarero et al., 2020). In general, these proposed explanations suggest that Gloger’s rule results from an increase in pigment load in feathers, stemming from selection on other traits. We observed that plumage brightness of both melanin and carotenoid-colored patches is negatively correlated with rainfall (though not with a measure of vegetation cover) as predicted by Gloger’s Rule, similar to what has been observed in Australian birds (Delhey, 2018) and in other American birds (Ramírez-Barrera et al., 2019). However, this contrasts with the results of Ribot et al. (2019), who found a strong association between plumage brightness and remotely sensed vegetation cover. Taken together, these results fail to support the hypothesis that darker plumage in this species is linked to a darker environment caused by denser vegetation coverage. An alternative explanation that is compatible with our results would be that the mechanism in play is selection on stronger sexual signaling and not increased crypsis. Somewhat counterintuitively, additional pigment load increases spectral purity (chroma) but decreases overall brightness, since pigments function by selective absorption of incoming light. As ambient light decreases, more pigment is necessary to achieve the same level of background contrast, but it also decreases the overall amount of light being reflected. Our data does not support the hypothesis that differences in plumage color are caused primarily by fixed genetic factors. A mutation which results in reduced function of CYP2J19-like genes or other genes involved in the carotenoid metabolism pathway would be unlikely to become fixed outside of founder effect scenarios, as these mutations appear to occur in very low frequencies in the wild and would be rather strongly selected against. We found that the color of males from the Tres Marias islands is significantly different from mainland males, and that this difference is strong enough to be distinguishable by avian visual systems. The color of Tres Marias males is achromatically brighter and less saturated compared to their mainland counterparts. This is consistent with the overall pattern we found, as the climatic conditions on the islands are more xeric than on the mainland, and the dominant vegetation presents a more open canopy. However, many island species across many taxa present reduced sexual signaling when compared to their mainland sister groups, and it has been hypothesized that this is due to changes in the intensity of sexual selection compared to the mainland.

We found no significant relationship between plumage brightness or red chroma and vegetation cover, which also shows limited support for the hypothesis that increase of red coloration is due to an increase in dietary carotenoid availability linked to the environment’s primary productivity as was the case in the work of Jacot et al. (2010). The strong positive correlation between feather color and rainfall suggests that the mechanism modulating carotenoid deposition is related to humidity, such as selection acting on immune response or mitochondrial activity. A direct measurement of mitochondrial activity or quantitative transcription of genes associated with immune response would allow us to directly test this hypothesis.

The lack of relationship between color variation and geographical distance indicates that the mechanism underlying this variation is not isolation by distance. This is in contrast to the findings of Núñez Zapata et al. (2018) and by Ramírez-Barrera et al. (2019), who used a similar approach and found that for *Turdus assimilis* and *Habia rubica* respectively, Isolation by Distance is the model that best explains color variation. This, coupled with the lack of genetic structure that correlates to geography suggests that there may be more gene flow than previously thought and/or that carotenoid-based coloration in this species is fairly plastic.

Coloration is a complex and likely highly modular phenotypic trait. While carotenoid-based coloration is undoubtedly under some degree of fixed genetic regulation, both experimental (*e.g.*, McGraw & Hill, 2000; Jacot et al., 2010) and comparative studies such as Ramírez-Barrera et al. (2019) and this one show that it is relatively plastic and subject to a large degree of environmental modulation. Our results show that a single gross-scale environmental measurement like mean annual rainfall can have a disproportionate effect on the coloration of certain taxa. However, the exact mechanisms that mediate the observed color differences have yet to be tested, and experimental studies that explicitly test these hypotheses are needed.

### Body size

Our results on body size are not conclusive; we found a statistically significant relationship between body size and geographic distance, suggesting that the mechanism driving body size variation is isolation by distance. This is similar to what was found in *Cranioleuca antisiensis*, where the mechanism underlying a dramatic change in body size between populations was found to be isolation by distance (Seeholzer & Brumfield, 2018). Similar results were also found by Ramírez-Barrera et al. (2019) in *Habia rubica*. These studies make apparent that variation in body size in these neotropical birds is mostly driven by geographic isolation. However, although the relationship we found was statistically significant, the effect size was very small and we would be cautious about extracting strong conclusions from it. A genomic scan of alleles linked to variation in body size would further clarify the role of genetic variation on phenotypic variation this species.

Taken together, our results show that coloration and body size evolution are seemingly decoupled in this species. In conclusion, we found that climate is an important driver of phenotypic divergence in this species. An experimental study manipulating availability of dietary carotenoids would help illuminate the proximate mechanisms underlying this divergence. Although our phylogenetic analysis shows that the two samples from Central America (ssp. *citrea*) separate into a well-supported clade, a more extensive sampling is required to clarify their taxonomic status.

Finally, although we find evidence of genetic structure in this taxon, we suggest that a broader sampling and more in-depth genomic analysis is necessary to fully assess the relationship between genomes, environment and phenotypes.

## Supplemental Information

10.7717/peerj.12901/supp-1 Supplemental Information 1 Suplemental Tables

10.7717/peerj.12901/supp-2 Supplemental Information 2 Supplemental Figures: Colorimetric and Morphometric analysis

## Additional Information and Declarations

### DNA Deposition

The following information was supplied regarding the deposition of DNA sequences: The sequences are available at Genbank SRA: BioProject accession PRJNA757783.

### Data Availability

The following information was supplied regarding data availability: The raw data is available at figshare: Robles Bello, Sahid Martin (2022): Piranga bidentata Raw Data. figshare. Dataset. https://doi.org/10.6084/m9.figshare.17096639.v1.
