# Supplementary material for: Drivers of phenotypic divergence in a Mesoamerican highland bird
Source: PeerJ. 2022 Feb 18;10:e12901. doi: 10.7717/peerj.12901 (PMC8860067; doi:10.7717/peerj.12901)
Supplement: Supplemental Information 1 [file peerj-10-12901-s001.docx]

|  | DF | Sum of Sq | Mean Sq | *F* | *p* |
| --- | --- | --- | --- | --- | --- |
| Brightness (B1) | 3 | 63914666 | 21304889 | 26.527 | **< 0.001** |
| Residuals (B1) | 71 | 57023111 | 803142 |  |  |
| Hue (H3) | 3 | 4661.6 | 1553.86 | 41.616 | **< 0.001** |
| Residuals (H3) | 71 | 2651 | 37.34 |  |  |
| Chroma (S8) | 3 | 2.067 | 0.68899 | 10.873 | **< 0.001** |
| Residuals (S8) | 71 | 4.4992 | 0.06337 |  |  |
| Red Chroma (S1R) | 3 | 0.079523 | 0.026508 | 13.374 | **< 0.001** |
| Residuals (S1R) | 71 | 0.140723 | 0.001982 |  |  |

**Table S1**. ANOVA table of male ventral color variables, with subspecies as the grouping variable. Statistically significant *p* values are shown in bold.

|  | DF | Sum of Sq | Mean Sq | *F* | *p* |
| --- | --- | --- | --- | --- | --- |
| Brightness (B1) | 3 | 34253559 | 11417853 | 7.7823 | **< 0.001** |
| Residuals (B1) | 46 | 67489384 | 1467161 |  |  |
| Hue (H3) | 3 | 192.68 | 64.228 | 3.3007 | 0.028 |
| Residuals (H3) | 46 | 895.1 | 19.459 |  |  |
| Chroma (S8) | 3 | 0.11929 | 0.039763 | 1.5239 | 0.22 |
| Residuals (S8) | 46 | 1.2003 | 0.026094 |  |  |

**Table S2**. ANOVA table of female ventral color variables, with subspecies as the grouping variable. Statistically significant *p* values are shown in bold.

|  | DF | Sum of Sq | Mean Sq | *F* | *p* |
| --- | --- | --- | --- | --- | --- |
| Culmen | 3 | 95.105 | 31.702 | 23.563 | **< 0.001** |
| Residuals (Culmen) | 141 | 141 | 1.345 |  |  |
| Exposed Culmen | 3 | 2.775 | 0.92514 | 1.1984 | 0.313 |
| Residuals (Exposed Culmen) | 141 | 108.845 | 0.77195 |  |  |
| Bill Width | 3 | 10.135 | 3.3783 | 32.998 | **< 0.001** |
| Residuals (Bill Width) | 140 | 14.333 | 0.1024 |  |  |
| Bill Depth | 3 | 15.504 | 5.1681 | 22.019 | **< 0.001** |
| Residuals (Bill Depth) | 134 | 31.451 | 0.2347 |  |  |
| Wing Chord | 3 | 297.37 | 99.122 | 7.8247 | **< 0.001** |
| Residuals (Wing Chord) | 148 | 1874.83 | 12.668 |  |  |
| Tarsus | 3 | 58.827 | 19.6091 | 28.32 | **< 0.001** |
| Residuals (Tarsus) | 147 | 101.787 | 0.6924 |  |  |

**Table S3**. ANOVA table of measured morphometric traits, with subspecies as the grouping variable. Statistically significant *p* values are shown in bold. Culmen was measured from the posterior edge of the nare to the beak tip, Exposed Culmen was measured from the edge of the last feathers to the beak tip, Bill Width was measured as the horizontal dimension of the beak at the nares, Bill Depth was measured as the vertical dimension of the beak at the nares. Wing Chord was measured from the ‘wrist’ joint to the tip of the longest primary feather, and Tarsus was measured from the notch of the tibio-tarsal joint to the last undivided tarsal scale.

| Subspecies | Culmen | Exposed Culmen | Beak Width | Beak Depth | Wing Cord | Tarsus Length |
| --- | --- | --- | --- | --- | --- | --- |
| *P. b. bidentata* | 14.3 ± 1.36 | 21.2 ± 0.78 | 8.09 ± 0.33 | 8.78 ± 0.48 | 97.6 ± 4.30 | 21.6 ± 0.83 |
| *P. b. citrea* | 13.0 ± 0.61 | 21.3 ± 1.21 | 8.03 ± 0.36 | 8.66 ± 0.48 | 93.4 ± 3.09 | 21.5 ± 1.07 |
| *P. b. flammea* | 15.6 ± 1.44 | 21.7 ± 0.57 | 8.73 ± 0.28 | 9.43 ± 0.44 | 95.1 ± 2.58 | 23.2 ± 0.47 |
| *P. b. sanguinolenta* | 14.1 ± 1.28 | 21.3 ± 0.70 | 7.86 ± 0.312 | 8.42 ± 0.51 | 96.3 ± 3.18 | 21.2 ± 0.83 |

**Table S4**. Summary of morphological measurements (mean ± standard deviation). Measures are in mm.

| Subspecies | Brightness (B1) | Chroma (S8) | Hue (H3) | Red Chroma (S1R) |
| --- | --- | --- | --- | --- |
| *P. b. bidentata* | 7561.79 ± 1076.86 | 2.04 ± 0.2 | 558.75 ± 4.96 | 0.52 ± 0.03 |
| *P. b. citrea* | 5666.24 ± 795.61 | 2.35 ± 0.27 | 575.54 ± 5.01 | 0.58 ± 0.05 |
| *P. b. flammea* | 8148.67 ± 913.18 | 2.07 ± 0.13 | 558.44 ± 8.11 | 0.52 ± 0.03 |
| *P. b. sanguinolenta* | 6125.95 ± 863.07 | 2.44 ± 0.29 | 575.17 ± 7 | 0.59 ± 0.05 |

**Table S5**. Summary of ventral (carotenoid) color variables in males (mean ± standard deviation).

| Subspecies | Brightness (B1) | Chroma (S8) | Hue (H3) |
| --- | --- | --- | --- |
| *P. b. bidentata* | 8634.76 ± 1800.54 | 1.66 ± 0.12 | 507 ± 4.21 |
| *P. b. citrea* | 6983.29 ± 1139.26 | 1.75 ± 0.17 | 511.92 ± 4.23 |
| *P. b. flammea* | 9525.42 ± 637.36 | 1.76 ± 0.05 | 507.5 ± 5.07 |
| *P. b. sanguinolenta* | 7272.37 ± 1005.87 | 1.81 ± 0.19 | 511.25 ± 4.73 |

**Table S6**. Summary of ventral (carotenoid) color variables in females (mean ± standard deviation).

| Subspecies | Brightness (B1) | Chroma (S8) | Hue (H3) |
| --- | --- | --- | --- |
| *P. b. bidentata* | 3599.33 ± 624.44 | 0.91 ± 0.19 | 571.75 ± 102.46 |
| *P. b. citrea* | 2766.37 ± 829.75 | 0.89 ± 0.19 | 582.92 ± 95.36 |
| *P. b. flammea* | 3694.42 ± 519.47 | 0.75 ± 0.15 | 610.67 ± 12.12 |
| *P. b. sanguinolenta* | 2951.25 ± 637.54 | 0.99 ± 0.16 | 610.38 ± 15.45 |

**Table S7**. Summary of tail (melanin) color variables in males (mean ± standard deviation).

| Subspecies | Brightness (B1) | Chroma (S8) | Hue (H3) |
| --- | --- | --- | --- |
| *P. b. bidentata* | 4384.08 ± 698 | 0.88 ± 0.19 | 491.12 ± 151.62 |
| *P. b. citrea* | 3199.61 ± 860.94 | 0.98 ± 0.15 | 571.19 ± 98.27 |
| *P. b. flammea* | 3281.72 ± 534.9 | 1.03 ± 0.23 | 597.75 ± 13.43 |
| *P. b. sanguinolenta* | 3706.19 ± 847.94 | 1.11 ± 0.18 | 591.58 ± 12.79 |

**Table S8**. Summary of tail (melanin) color variables in females (mean ± standard deviation).
