# Supplementary material for: Drivers of phenotypic divergence in a Mesoamerican highland bird
Source: PeerJ. 2022 Feb 18;10:e12901. doi: 10.7717/peerj.12901 (PMC8860067; doi:10.7717/peerj.12901)
Supplement: Supplemental Information 2 [file peerj-10-12901-s002.docx]

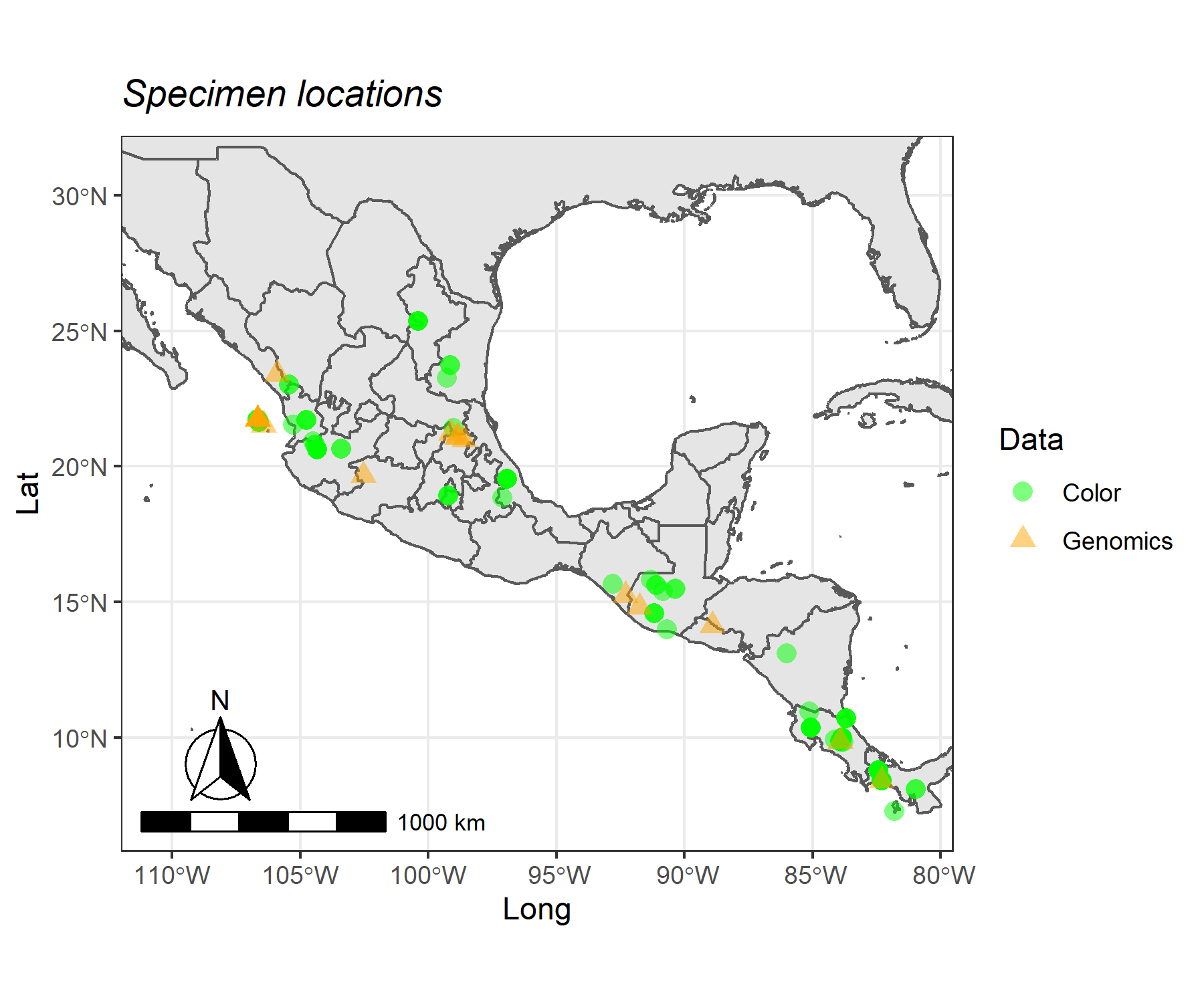


**Figure S1**. Geographic location of studied samples. Samples in green are specimens from which we could only obtain color data, samples in yellow are specimens from which we have genomic data.


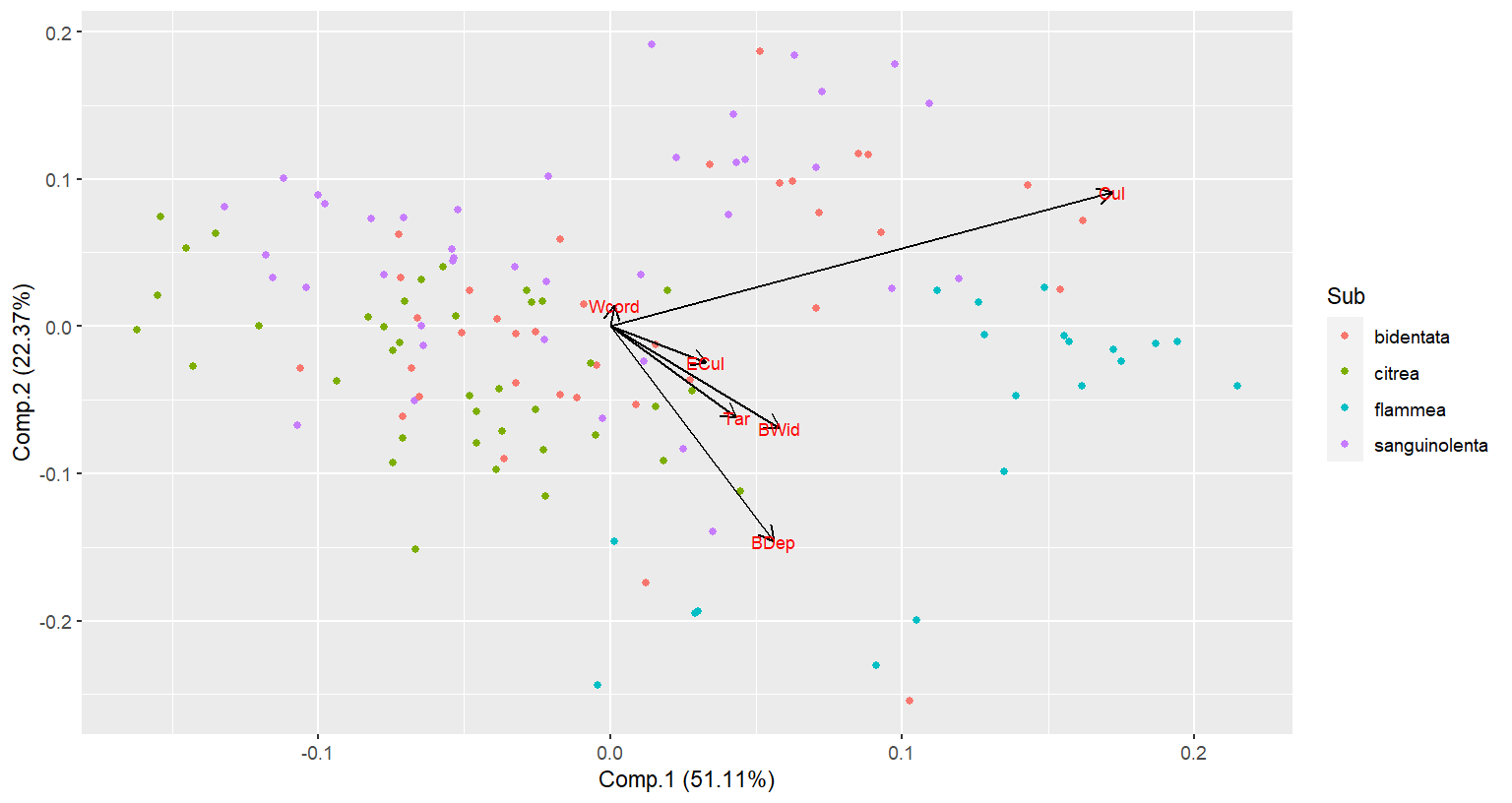


**Figure S2**. PCA of morphological variables. The first two principal components explain 73.48% of variation.
